# Roles of the Host Oxidative Immune Response and Bacterial Antioxidant Rubrerythrin during Porphyromonas gingivalis Infection 

**DOI:** 10.1371/journal.ppat.0020076

**Published:** 2006-07-28

**Authors:** Piotr Mydel, Yusuke Takahashi, Hiromichi Yumoto, Maryta Sztukowska, Malgorzata Kubica, Frank C Gibson, Donald M Kurtz, Jim Travis, L. Vincent Collins, Ky-Anh Nguyen, Caroline Attardo Genco, Jan Potempa

**Affiliations:** 1 Department of Microbiology, Faculty of Biochemistry, Biophysics, and Biotechnology, Jagiellonian University, Krakow, Poland; 2 Department of Medicine, Boston University School of Medicine, Boston, Massachusetts, United States of America; 3 Department of Oral Microbiology, Kanagawa Dental College, Yokosuka, Kanagawa, Japan; 4 Department of Conservative Dentistry, The University of Tokushima, School of Dentistry, Tokushima, Japan; 5 Department of Biochemistry and Molecular Biology, University of Georgia, Athens, Georgia, United States of America; 6 Department of Chemistry and Center for Metalloenzyme Studies, University of Georgia, Athens, Georgia, United States of America; 7 Department of Rheumatology and Inflammation Research, University of Göteborg, Göteborg, Sweden; University of California Berkeley, United States of America

## Abstract

The efficient clearance of microbes by neutrophils requires the concerted action of reactive oxygen species and microbicidal components within leukocyte secretory granules. Rubrerythrin (Rbr) is a nonheme iron protein that protects many air-sensitive bacteria against oxidative stress. Using oxidative burst-knockout (NADPH oxidase–null) mice and an *rbr* gene knockout bacterial strain, we investigated the interplay between the phagocytic oxidative burst of the host and the oxidative stress response of the anaerobic periodontal pathogen *Porphyromonas gingivalis.* Rbr ensured the proliferation of P. gingivalis in mice that possessed a fully functional oxidative burst response, but not in NADPH oxidase–null mice. Furthermore, the in vivo protection afforded by Rbr was not associated with the oxidative burst responses of isolated neutrophils in vitro. Although the phagocyte-derived oxidative burst response was largely ineffective against P. gingivalis infection, the corresponding oxidative response to the Rbr-positive microbe contributed to host-induced pathology via potent mobilization and systemic activation of neutrophils. It appeared that Rbr also provided protection against reactive nitrogen species, thereby ensuring the survival of P. gingivalis in the infected host. The presence of the *rbr* gene in P. gingivalis also led to greater oral bone loss upon infection. Collectively, these results indicate that the host oxidative burst paradoxically enhances the survival of P. gingivalis by exacerbating local and systemic inflammation, thereby contributing to the morbidity and mortality associated with infection.

## Synopsis

The physiological role of neutrophils is to seek out and destroy invading microbes. Professional phagocytes engulf (phagocytose) these organisms and kill them using bactericidal peptides, enzymes, toxic reactive oxygen species, and reactive nitrogen species produced by neutrophils and macrophages. Unfortunately, the reactive oxygen species unleashed in an oxidative burst response can cause considerable collateral damage and are directly responsible for infection-associated tissues injuries, especially if the invaders are protected against killing by neutrophils. The authors investigated the pathogenesis of *Porphyromonas gingivalis,* an anaerobic bacterium that is responsible for human periodontal disease and is protected against oxidative stress by the cytoplasmic protein rubrerythrin. We show that P. gingivalis is not only resistant to reactive oxygen species, but that in mice, rubrerythrin shields the bacterium against reactive nitrogen species. These features allow P. gingivalis to proliferate in animals that possess a fully functional oxidative burst response. Furthermore, we demonstrate that the neutrophil oxidative burst response, rather than eliminating the bacteria, exacerbates disease by damaging host tissues and facilitating growth and systemic dissemination of the pathogen. Collectively, this study provides important information on how oxygen-dependent killing mechanisms operate during anaerobic infection and on the role of rubrerythrin in protecting against a pathogenic anaerobic organism, while emphasizing the importance of limiting host-mediated tissue injury in inflammatory diseases caused by bacteria.

## Introduction

Phagocytic leukocytes, especially neutrophils, play a critical role in innate immune responses against bacteria, fungi, and other pathogens [1]. Neutrophil-mediated bacterial killing can involve both oxygen-independent and oxygen-dependent processes. Oxygen-independent microbial killing relies on the contents of three cytoplasmic granule subsets, the azurophilic (primary), specific (secondary), and gelatinase granules. After fusing with phagosomes, these granules deliver antimicrobial proteins and peptides, such as defensins, bactericidal/permeability-increasing protein, azurocidin, cathelicidin, and lysozyme, all of which are capable of damaging the bacterial cell envelope. In addition, several proteinases, such as neutrophil elastase and cathepsin G, facilitate bacterial killing by digestion of bacterial outer membrane proteins [2], surface appendages [3], and virulence factors [4]. Oxygen-independent microbial killing also appears to function efficiently extracellularly. Upon activation, neutrophils release granule proteins and chromatin, which together form extracellular fibers. Brinkman et al. [5] have reported that these fibers become saturated with high concentrations of bactericidal peptides, proteins, and proteases, and are able to trap and efficiently kill invading bacteria that become entangled in the fiber meshwork. The significance of this oxygen-independent mechanism is exemplified by recurrent infections associated with two rare inherited diseases, Chediak-Higashi syndrome and specific granule deficiency, which are characterized by insufficient release of antimicrobial components and the absence of some antimicrobial components in specific and/or azurophilic granules, respectively [6].

Oxygen-dependent killing is initiated by the assembly of the NADPH oxidase complex at the phagosome membrane [7]. The NADPH oxidase system is required for immunocompetence against bacteria via the so-called respiratory or oxidative burst, which generates reactive oxygen species (ROS) that are toxic for microbes [8,9]. The importance of this oxygen-dependent killing is exemplified by patients with chronic granulomatous disease (CGD), who carry a dysfunctional NADPH oxidase in their phagocytes and often suffer from recurrent infections [8]. However, patients with CGD rarely have infections that involve anaerobic bacteria [2,6,10,11], which suggests that oxygen-dependent bacterial killing mechanisms are not utilized for the clearance of anaerobic organisms. Furthermore, recent studies have demonstrated a link between the formation of superoxide anion and the activation of granular microbicidal enzymes, which suggests a new paradigm for the molecular mechanisms utilized by neutrophils to kill their targets [12,13]. Thus, the oxidative burst is a prerequisite for the mobilization of cationic neutrophil elastase and cathepsin G, which are stored in granules complexed with the strongly anionic sulfated proteoglycan matrix. In addition, one of the major bactericidal peptides of neutrophils is released from cathelicidins by the action of neutrophil proteases [14]. Thus, it is clear that the elimination of invading bacteria by phagocytes is a complicated and highly coordinated process.

The importance of the orchestrated activation and recruitment of neutrophils for removing bacterial pathogens is clearly evident in the oral cavity during periodontal disease, where the innate immune system responds to an abundant and versatile microflora that matches in mass and complexity those residing in the lower gastrointestinal tract. The tenuous balance between healthy and diseased states within the gingival sulcus is maintained primarily by neutrophils. Even minor, inherited, or acquired malfunctions of neutrophils, but not of other leukocytes, invariably result in severe inflammation of the gingiva (gingivitis), while more pronounced defects in phagocyte function predispose to rapid destruction of the periodontium (manifested as periodontal disease) and subsequent tooth loss [10,15,16]. The onset and progression of periodontal disease is associated with the proliferation of specific bacterial species, particularly anaerobes, which include *Porphyromonas gingivalis, Haemophilus (Actinobacillus) actinomycetemcomitans, Treponema denticola,* and *Tannerella (Bacteroides) forsythensis* [17]. P. gingivalis has been implicated as the major pathogen associated with adult periodontal disease. In addition, this organism is also associated with localized aggressive periodontitis, which is a more aggressive and destructive form of periodontal disease associated with impaired neutrophil chemotaxis [18]. Patients with localized aggressive periodontitis are otherwise in good general health and are not predisposed to extraoral microbial infections, further supporting the crucial importance of fully functional neutrophils in maintaining homeostasis in the gingival tissues.

P. gingivalis possesses an array of virulence factors, including lipopolysaccharide (LPS), fimbriae, hemagglutinins, hemolysins, and proteolytic enzymes, which facilitate colonization and the initiation of infection [19]. P. gingivalis also contains enzymes that have been implicated in protection against oxidative stress, such as an excision/repair system for removing oxidatively damaged DNA bases [20], superoxide dismutase [21], and rubrerythrin (Rbr) [22]. Since the P. gingivalis strain that lacks the gene for rubrerythrin *(rbr^−^)* is more dioxygen sensitive and hydrogen peroxide sensitive than the wild-type strain and shows growth inhibition in liquid cultures when exposed to oxidants, it has been proposed that Rbr protects *P. gingivalis* against aerobic stress by functioning as a cytoplasmic peroxidase that reduces hydrogen peroxide to water [22]. Rbr appears to be involved in protection against oxidative stress in several anaerobic bacteria and archaebacteria, most likely functioning as the terminal component of an NAD(P)H peroxidase [23–25]. In addition, P. gingivalis accumulates a hemin layer that is purported to provide oxidative stress protection [26].

The manner in which these bacterial oxidative stress protection systems function to protect anaerobic organisms against the oxygen-dependent bactericidal activity of neutrophils has not been well defined. Using P. gingivalis as a model organism for anaerobic infection, we initially investigated the role of NADPH oxidase–dependent mechanisms in the responses of mice to infection with anaerobic bacteria. Subsequently, we examined the contribution of Rbr to the protection of this anaerobic organism against the host oxidative burst. Our results indicate that the host oxygen-dependent bactericidal system is not only ineffective in combating P. gingivalis infection, but appears to exacerbate significantly the host tissue damage induced by the infection. Furthermore, we show that Rbr plays an important protective role for the bacteria in the presence of a fully functional host immune response. This function of Rbr is unrelated to the neutrophil oxidative burst and is manifested as the shielding of P. gingivalis against reactive nitrogen species (RNS).

## Results

### Rbr Is Important for P. gingivalis Virulence

**Figure 1:**
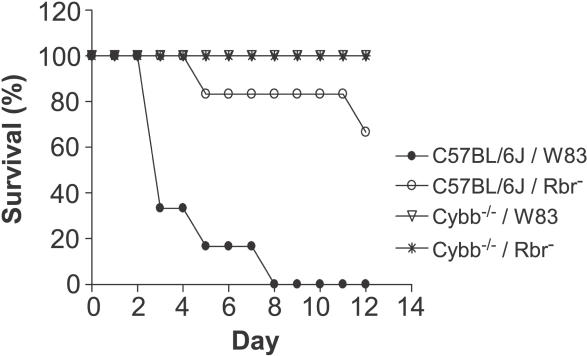
Viability of Mice Inoculated with P. gingivalis The data shown are the percentages of mice (*n* = 7 for each group) that were alive on the indicated days after challenge with 100-μl volume, 1 × 10^9^ CFU P. gingivalis wild-type (W83) or Rbr mutant (Rbr^−^). Control animals were inoculated with sterile broth. Wild-type (C57BL/6J) and Cybb^−/−^ mice were utilized.

Since Rbr protects P. gingivalis against oxidative stress in vitro [22], we investigated whether it would protect P. gingivalis against oxidative stress during infection in an animal model. For these initial studies, we utilized a mouse subcutaneous chamber model in which virulent strains of the bacterium were able to disseminate from an initial infection site and cause high mortality and morbidity [27,28]. Subcutaneous chambers implanted into C57BL/6J mice were inoculated with either a lethal dose (100-μl volume, 1 × 10^9^ CFU/ml) of P. gingivalis W83 or the same dosage of the isogenic Rbr^−^ strain, and the mice were monitored daily for general health condition and the emergence of skin abscesses. Mice infected with the wild-type P. gingivalis strain exhibited severe cachexia, with ruffled hair, hunched bodies, and weight loss, as well as severe skin lesions localized on the ventral body surface. All seven of the animals infected with wild-type P. gingivalis died by day 8 after infection (Figure 1). In contrast, two of the seven mice that were inoculated with the P. gingivalis Rbr^−^ strain succumbed to infection on days 5 and 12 after infection, while the five remaining mice appeared healthy and resembled the unchallenged animals.

**Figure 2:**
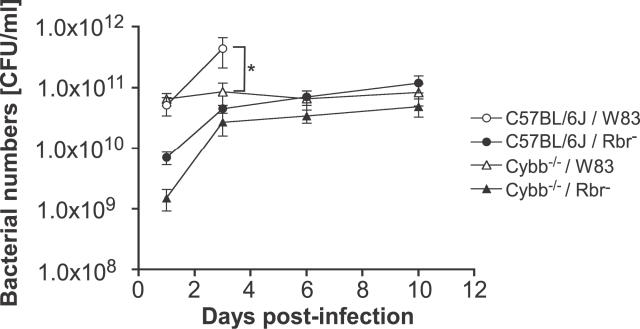
Bacterial Viability in Chamber Fluid Samples Subcutaneous chambers were inoculated on day 0 with 100-μl volume, 1 × 10^9^ CFU/ml of the P. gingivalis strains. The CFU/ml values were determined by counting bacterial colonies on anaerobic agar plates. *p < 0.001 for data on day 3; Mann-Whitney *U* test (*n* = 7 mice for each group). Increased numbers of P. gingivalis were detected on the third day in C57BL/6J mice inoculated with strain W83 (wild-type) as compared to the Cybb^−/−^ mice and challenge with the *P. gingivalis* Rbr^−^ strain.

To investigate whether the higher survival rates of animals inoculated with the P. gingivalis Rbr^−^ strain were the result of altered bacterial growth rates in vivo, the bacterial numbers in the chambers were determined on days 1, 3, 6, and 10 following bacterial challenge. Following infection with the P. gingivalis Rbr^−^ strain, the number of organisms recovered from the chamber fluid samples gradually increased to a level on day 10 after inoculation that was approximately 100-fold higher than that of the original inoculum. The numbers of bacteria in the samples collected from the chambers of C57BL/6J mice infected with the wild-type P. gingivalis strain increased ~50-fold on day 1, increased further to 3.5 × 10^11^ CFU/ml on day 3, and reached a level that was 300-fold higher than that administered initially (100-μl volume, 1 × 10^9^ CFU/ml) (Figure 2). These data indicate that Rbr expression is important for P. gingivalis proliferation in subcutaneous chambers.

### Oxidative Burst Does Not Contribute to the Killing of P. gingivalis by Human or Mouse Neutrophils

**Figure 3:**
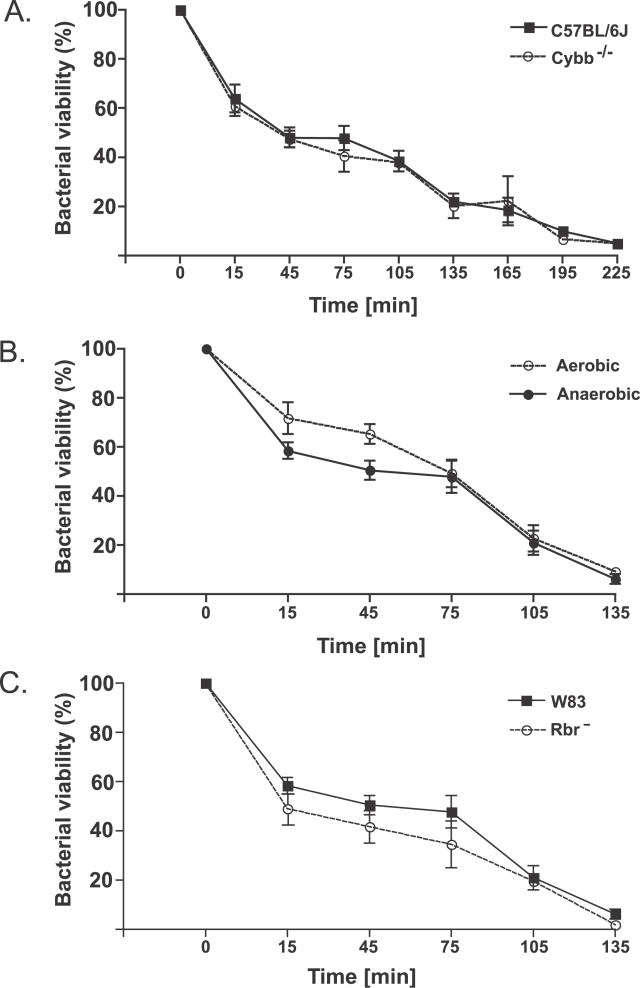
P. gingivalis Killing by Neutrophils Murine bone marrow-derived neutrophils isolated from wild-type and Cybb^−/−^ animals were incubated with opsonized P. gingivalis (wild-type or Rbr^−^ strain) at a ratio of 1:5 ratio in an aerobic or anaerobic atmosphere. At the indicated timepoints, samples were withdrawn and plated, and the numbers of bacteria were determined. The CFU value at time zero was taken as 100%. (A) Wild-type P. gingivalis incubated aerobically with neutrophils derived from wild-type (C57BL/6J) and Cybb^−/−^ mice. (B) Wild-type P. gingivalis incubated aerobically or anaerobically with neutrophils from wild-type (C57BL/6J) mice. (C) Wild-type P. gingivalis and the Rbr^−^ strain incubated aerobically with neutrophils from wild-type (C57BL/6J) mice.

To investigate the contribution of the neutrophil oxidative burst to the elimination of *P. gingivalis,* we compared the rates of killing of this bacterium by neutrophils that were isolated from wild-type and NADPH oxidase–null (Cybb^−/−^) mice. Surprisingly, the rate of P. gingivalis killing by normal bone marrow murine neutrophils was indistinguishable from that by oxidative burst-deficient neutrophils. The killing by both cell types was relatively slow, with 50% and 10% viable bacterial cells persisting after 45 and 195 min of coincubation with neutrophils, respectively (Figure 3A). Significantly, except for the shorter periods of incubation, there was no difference in the rates of killing of the wild-type P. gingivalis by the normal mouse neutrophils under oxic and anoxic conditions (Figure 3B), where the production of ROS has been reported to be diminished under anoxic conditions [29]. From a comparison of the killing of P. gingivalis wild-type and Rbr^−^ strains under aerobic conditions (Figure 3C), it is clear that the elimination of viable bacteria occurs with the same kinetics regardless of the presence or absence of Rbr. The human peripheral blood neutrophils killed P. gingivalis more efficiently than the mouse neutrophils, but again, there was no difference between the rates of killing of the wild-type and Rbr^−^ strains (data not shown). Taken together, our results indicate that Rbr does not contribute to the resistance of P. gingivalis to the oxygen-dependent bactericidal activity of neutrophils and that the host oxidative burst does not play a major role in the elimination of P. gingivalis. These data corroborate the clinical observation that patients with CGD are not unusually susceptible to periodontal diseases despite the lack of oxygen-dependent bactericidal activities of their neutrophils [10], and support the hypothesis that antibacterial peptides and proteins potentially constitute the major armamentarium used by neutrophils to kill P. gingivalis [30,31].

### Cybb^−/−^ Mice Are Resistant to P. gingivalis Infection

To investigate further the involvement of the oxygen-dependent antimicrobial defense mechanism against systemic infection by anaerobic bacteria, we evaluated the susceptibility of Cybb^−/−^ mice to intraperitoneal chamber inoculation with a lethal dose (100-μl volume, 1 × 10^9^ CFU/ml) of *P. gingivalis.* Interestingly, we found that Cybb^−/−^ mice were resistant to infection with either the wild-type or P. gingivalis Rbr^−^ strains, as evidenced by the survival of all the mice following systemic challenge up to the end of the experiment (Figure 1). Correlating this difference in survival relative to that seen for C57BL/6J mice, a distinct difference in the kinetics of in vivo bacterial growth was observed between the C57BL/6J and Cybb^−/−^ mice. In the C57BL/6J mice, an abrupt increase in bacterial numbers occurred 1 d after infection with P. gingivalis W83, and the bacterial numbers increased further by day 3 (Figure 2), at which point ~70% of the animals had died (Figure 1). Conversely, in Cybb^−/−^ mice, although the bacterial numbers reached the same level as in the wild-type animals on day 1, the bacterial numbers did not increase during the subsequent days of infection (Figure 2).

The P. gingivalis Rbr^−^ strain was detected in lower numbers than the wild-type strain in the chamber fluid samples at day 1. Following a significant increase from day 1 to day 3, the number of Rbr^−^ bacteria within the chamber fluid samples remained constant over the 10-d infection period (Figure 2). Despite the increased sensitivity of the Rbr^−^ strain to oxidative stress in vitro [22], it seems unlikely that the lack of Rbr is responsible for the retarded growth of the mutant strain in vivo since P. gingivalis is apparently killed by neutrophils in an oxygen-independent manner, for which Rbr has no protective function. Furthermore, the presence of Rbr does not affect elimination of the bacterium by fully functional neutrophils that respond to the phagocytosed microbes.

### RNS Contribute to P. gingivalis Killing by Macrophages, and Rbr Exerts a Protective Effect against iNOS-Dependent Bactericidal Activity

**Figure 4:**
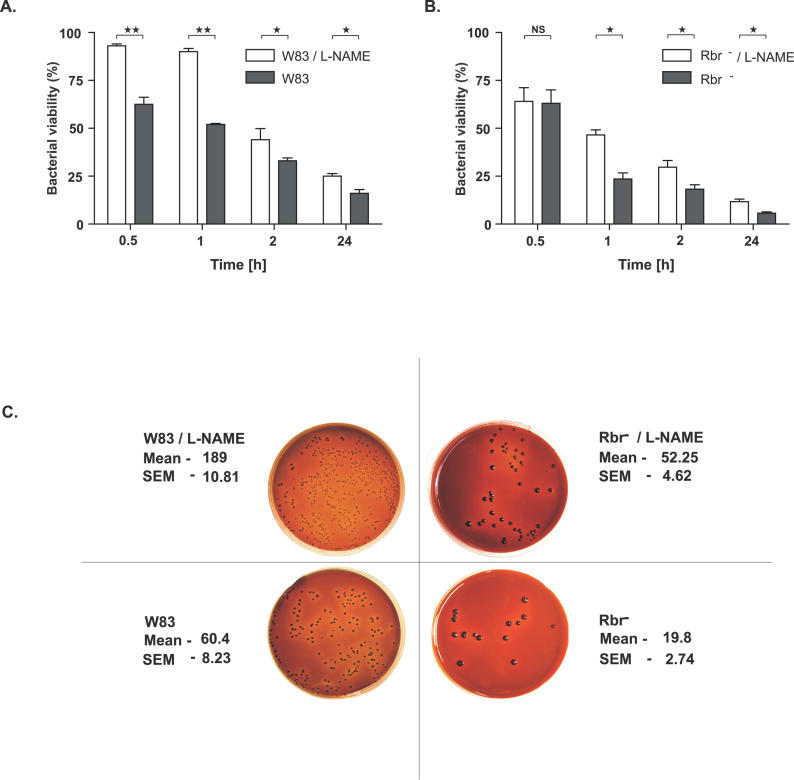
P. gingivalis Killing by Murine Macrophages in the Presence and Absence of Nitric Oxide Murine macrophages were incubated with opsonized P. gingivalis (the wild-type or Rbr^−^ strain) at a ratio of 1:10 in the presence or absence of iNOS inhibitor (L-NAME). At the indicated timepoints, samples were withdrawn and plated, and the numbers of bacteria were determined. The CFU value at time zero was taken as 100%. (A) Wild-type P. gingivalis incubated with macrophages in the presence or absence of L-NAME. (B) Rbr^−^ strain incubated with macrophages in the presence or absence of L-NAME. (C) Representative pictures of P. gingivalis W83 (left panel) and Rbr^−^ (right panel) colonies from the 24-h timepoint grown on anaerobic blood agar plates. In (A) and (B), statistical significance of the differences in the CFU values was calculated using the Mann-Whitney *U* test (*p < 0.05; **p < 0.01).

The different kinetics of Rbr^−^ and wild-type P. gingivalis growth in vivo (Figure 2) suggests that Rbr provides protection against the oxygen-independent antimicrobial activity of the host. This activity is elicited mainly by either bactericidal peptides/proteins or RNS. RNS are generated in macrophages, monocytes, and epithelial cells by inducible nitric oxide synthase (iNOS) in response to bacterial infection. Therefore, we compared the susceptibilities of the P. gingivalis wild-type strain and Rbr mutant to killing after phagocytosis by explanted mice peritoneal macrophages in the absence or presence of the specific iNOS inhibitor L-NAME. Of note, L-NAME at the concentration used did not affect proteolytic activity of gingipains R, Arg-Xaa peptide bond–specific proteases, which are considered the major virulence factor of *P. gingivalis.* The significantly higher rates of killing of the wild-type strain (Figure 4A) and the Rbr^−^ mutant (Figure 4B) by macrophages with active iNOS underline the importance of RNS in P. gingivalis elimination and indicate that Rbr provides protection against RNS. This conclusion is validated by a direct comparison of the bacterial CFU in the macrophage lysates after 24 h of phagocytosis (Figure 4C). In summary, this finding corroborates well with the observed in vivo differences in pathological changes in mice that were inoculated with the wild-type strain and Rbr mutant in normal and oxidative burst–deficient animals.

### Growth of Wild-Type P. gingivalis in C57BL/6J Mice Is Associated with the Release of Large Quantities of LPS

**Figure 5:**
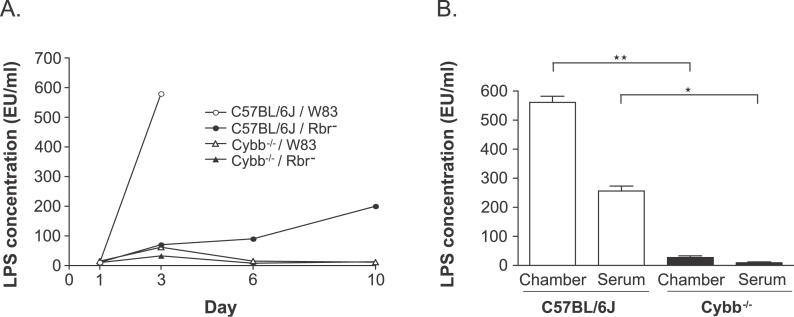
LPS Levels in Chamber Fluid Samples and/or Sera of C57BL/6J and Cybb^−/−^ Mice during the Course of the Experiment (A) LPS levels in the chamber fluid samples of C57BL/6J and Cybb^−/−^ mice. On day 0, the two mouse strains were inoculated with 100-μl volume, 1 × 10^9^ CFU/ml P. gingivalis strain W83 or Rbr^−^ mutant strain. (B) LPS levels in the chamber fluid samples and sera of mice on day 3 after challenge. The mice were challenged with a lethal dose (100-μl volume, 1 × 10^9^ CFU/ml) of P. gingivalis strain W83. The data are presented as the means ± SD from the results obtained for three mice per group. *p < 0.05; **p < 0.01; as determined by Student's *t* test.

The killing of Gram-negative bacteria in vivo may lead to the release of LPS [32], which can cause an aggravated septic shock. Therefore, we compared the levels of soluble LPS in the chamber fluids and sera of mice that were challenged with P. gingivalis (Figure 5). In the chamber fluid samples from C57BL/6J mice that were inoculated with the wild-type P. gingivalis strain, the concentration of LPS increased from the baseline (10 EU/ml) on day 1 to 600 EU/ml on day 3 (Figure 5A). Only moderately elevated levels of LPS (130 EU/ml) were detected in the chamber fluids from C57BL/6J mice that were infected with the Rbr^−^ mutant on days 3 and 6 after inoculation, and these levels increased further to 230 EU/ml on day 10. The LPS levels in the chamber fluid samples from the oxidative burst–deficient mice increased transiently on day 3 and returned to the baseline level by day 6 (Figure 5A). In addition, high levels of LPS were detected in the sera of C57BL/6J mice inoculated with the wild-type P. gingivalis strain (Figure 5B). In the case of Cybb^−/−^ mice inoculated with the wild-type strains of P. gingivalis (Figure 5B) or C57BL/6J mice inoculated with the Rbr^−^ strain, the concentrations of LPS in the sera did not differ significantly from the background levels (unpublished data). These results suggest that the respiratory burst is responsible for LPS release from bacteria, both in the chamber fluid samples and in infected tissues, and especially in the blood.

### Lack of Cytokines in the Chamber Fluid Samples Following P. gingivalis Infection

We next evaluated the levels of the proinflammatory cytokines TNF-α and IL-6 in chamber fluid and serum samples following challenge with *P. gingivalis.* Surprisingly and contrary to a previous report [33], no significant concentrations of these cytokines were detected in the chamber fluid samples following infection with either the wild-type or the Rbr^−^ strain (unpublished data). This discrepancy can be attributed to the degradation of cytokines by cysteine proteases (gingipain R and gingipain K) produced by *P. gingivalis.* Several studies have shown that IL-1β, TNF-α, and IL-6 are efficiently degraded by gingipains, both in vivo and in vitro [34–36]. This assumption is supported by the high levels of gingipain R and gingipain K activity seen in the chamber fluid samples from P. gingivalis–infected mice. We also found that the levels of gingipain activity correlated well (*p* < 0.005) with CFU counts (unpublished data), confirming the production of gingipains in vivo.

### Systemic Cytokine Responses to P. gingivalis Infection Do Not Correlate with Levels of LPS Release

**Figure 6:**
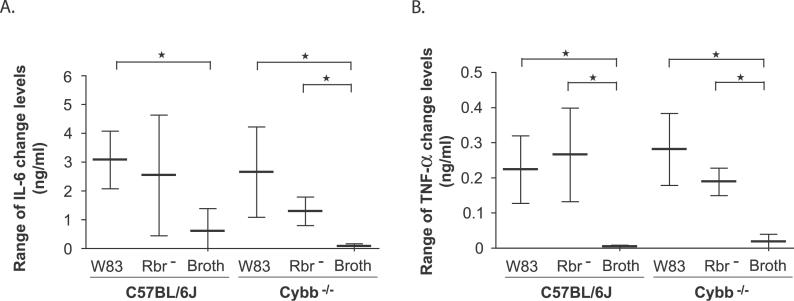
Cytokine Levels in the Sera of C57BL/6J and Cybb^−/−^ Mice after Challenge with the *P. gingivalis* Wild-Type (W83), the Rbr Mutant (Rbr^−^) Strain, and Sterile, Noninoculated Anaerobic Broth (Broth) The subcutaneous chambers in the wild-type (C57BL/6J) and Cybb^−/−^ mice were inoculated with 100-μl volume, 1 × 10^9^ CFU/ml P. gingivalis or with broth on day 0. The IL-6 (A) and TNF-α (B) levels were measured 1, 3, 6, and 10 d after inoculation. No statistically significant differences in cytokine levels are evident between the wild-type and Cybb^−/−^ mice following P. gingivalis challenge. The data shown are the median and interquartile range for each group of mice (*n* = 7) throughout the course of the experiment. Comparisons are made using the Mann-Whitney *U* test (*p* < 0.05).

We measured the levels of specific cytokines in serum samples obtained from P. gingivalis–infected mice. With the exception of IL-6 in wild-type mice inoculated with *P. gingivalis,* both the IL-6 and TNF-α levels increased significantly above the baseline levels in mice of either genotype infected with either the wild-type or the Rbr^−^ P. gingivalis strain (Figure 6). However, there was no correlation between the cytokine responses and LPS concentrations (unpublished data). Cybb^−/−^ mice inoculated with the P. gingivalis strain or normal mice infected with the Rbr^−^ mutant, although showing a background level of LPS in the serum (Figure 5), elicited similar amounts of IL-6 (Figure 6A) and TNF-α (Figure 6B) compared with normal mice inoculated with the wild-type strain and having a high concentration of LPS in the serum. These results suggest that the increased cytokine production in animals challenged with P. gingivalis is at least partially independent of LPS release.

### Wild-Type and Rbr^−^ P. gingivalis Strains Elicit a Cellular Inflammatory Response

**Figure 7:**
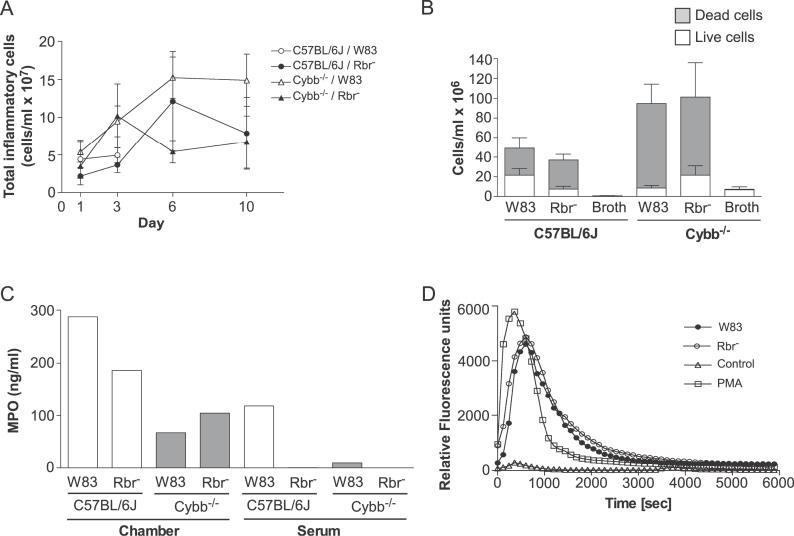
Inflammatory Cell Infiltrates in Chamber Fluid Samples of Mice Challenged with P. gingivalis (A) The total cell counts are similar between the groups (*n* = 7), as determined by trypan blue staining and cell counting (*p* > 0.05 for the course of the experiment; Mann-Whitney *U* test). In both wild-type and Cybb^−/−^ mice, the cell numbers in the chambers inoculated with the sterile P. gingivalis cultivation broth were similar (0.5 × 10^7^ cell/ml) throughout the course of the experiment (not shown in the figure). (B) The numbers of live and dead cells in the chamber fluid on day 3 after inoculation. (C) MPO was measured by ELISA. The chamber fluid and serum MPO levels in the wild-type P. gingivalis–infected mice on day 3 after inoculation are approximately four times higher than the corresponding levels in the P. gingivalis Rbr^−^-inoculated animals (*n* = 7, pooled samples). Control animals inoculated with sterile, noninoculated anaerobic broth had very low levels of MPO (data not shown). (D) Both the wild-type and Rbr-deficient strains of P. gingivalis cause strong activation of the NADPH oxidase in the mouse bone marrow neutrophils. No statistically significant differences were noted between the strains.

In agreement with previous studies [27,28,33], we observed that challenging mice with P. gingivalis via subcutaneously implanted chambers elicited a robust cellular inflammatory response. In addition, we found that the total leukocyte counts recovered in chamber fluid samples were similar and remained at the same level throughout the experiment, regardless of the mouse genotype or the strain of P. gingivalis used (Figure 7A). In corroboration of other reports [27,28,33], the differential cell count analysis indicated that the predominant inflammatory cells in the chamber fluid samples following P. gingivalis challenge were neutrophils. We did not observe significant differences, either qualitatively or quantitatively, in the levels of infiltration of inflammatory cells into the chambers between C57BL/6J and Cybb^−/−^ mice regardless of whether they were challenged with the wild-type or Rbr^−^ strain (unpublished data). In addition, within each group of animals the numbers of live and dead cells observed 3 days after bacterial challenge were similar, regardless of the strain used for inoculation (Figure 7B). Nonetheless, in the case of C57BL/6J mice inoculated with wild-type *P. gingivalis,* the influx of neutrophils was apparently insufficient to contain the infection.

Since myeloperoxidase (MPO) can be considered a marker of local or systemic neutrophil activation [37–48] and is a good indicator of the rate of neutrophil turnover, we determined the concentration of this enzyme in the chamber fluids and sera of mice inoculated with a lethal dose of wild-type *P. gingivalis.* As expected, MPO was present in the chamber fluid samples collected on day 3 after inoculation, with the enzyme concentration being four-fold higher in the samples obtained from the wild-type mice than from Cybb^−/−^ mice inoculated with wild-type P. gingivalis. In the case of inoculation with the *rbr^−^* strain, the difference in the MPO level in the chamber fluid between wild-type and Cybb^−/−^ mice was about two-fold (Figure 7C). It seems likely that the higher level of MPO in the chamber fluid from wild-type mice was not due to increased lysis of the neutrophils, since the numbers of dead leukocytes were much higher in Cybb^−/−^ mice than in wild-type mice (Figure 7B). This observation suggests that in the absence of oxidative burst, the increased influx of leukocytes into the chambers is followed by cell death.

**Figure 8:**
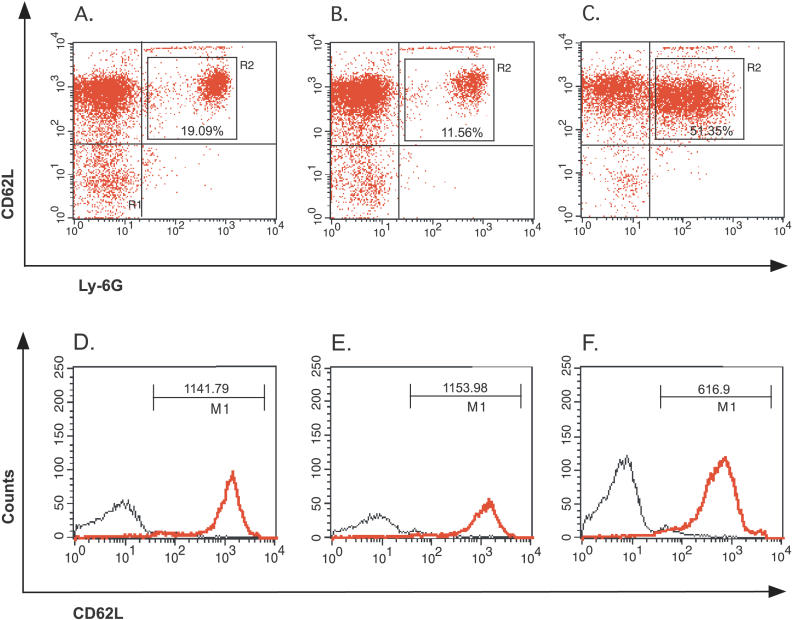
Challenge with P. gingivalis W83 Strain Elicits Potent Neutrophil Recruitment and Activation in the Peripheral Blood Compared to the Rbr^−^ Strain The top row (dotplots) shows the flow cytometric analysis of blood cells stained with Ly-6G and CD62L 3 d after chamber inoculation with (A) PBS, (B) the Rbr^−^ strain, and (C) the W83 strain. In the case of mice infected with the W83 strain, the number of neutrophils increased five-fold compared to the number of neutrophils in mice infected with the Rbr^−^ strain (gated events). The histograms in the bottom row exhibit a two-fold decrease in CD62L expression for peripheral blood neutrophils collected from (F) W83-challenged mice in comparison to (D) control and (E) the *rbr^−^* mutant strain–infected animals. Black line, isotypic control.

Significant quantities of MPO were detected exclusively in the serum samples from wild-type mice inoculated with wild-type P. gingivalis (Figure 7C). This observation suggests that systemic activation of neutrophils or increased turnover of these cells is caused only by the wild-type bacteria present in the circulation of wide-type mice. Notably, both the *rbr^−^* mutant and the parental P. gingivalis strain triggered the same level of NADPH oxidase activation in murine bone marrow neutrophils (Figure 7D). Therefore, to elucidate further the profound differences in the host responses to P. gingivalis strains, as illustrated by the serum MPO levels, we compared using flow cytometry analysis of the status of neutrophils in the whole blood of C57BL/6J mice that were infected with either the wild-type or the *rbr^−^ P. gingivalis* strain. Staining the blood for Ly-6G, which is an antigen that is expressed constitutively on mice leukocytes [49], revealed an approximately 2.5-fold increase in neutrophils in animals infected with P. gingivalis W83 compared to the control (uninfected) mice or those inoculated with the *rbr^−^* mutant (Figure 8). Furthermore, neutrophils from mice infected with wild-type P. gingivalis presented significantly less L-selectin, a marker for neutrophil activation, on their surfaces than neutrophils from the other groups of animals (Figure 8). Collectively, the flow cytometry data corroborate the MPO assay results and indicate that mouse infection with wild-type *P. gingivalis,* but not with the *rbr^−^* strain, triggers an increase in the number of circulating neutrophils and boosts the systemic activation of these cells. In the bloodstream, the encounter between wild-type P. gingivalis and neutrophils that have a functional oxidative burst probably leads to the release of large quantities of LPS (Figure 5). It is tempting to speculate that in addition to disseminated infection and colonization of other organs (see below) systemic activation of neutrophils was responsible for the observed mouse mortality.

### Impaired Clearance of P. gingivalis from Chambers Leads to Systemic Dissemination of the Infection

**Figure 9:**
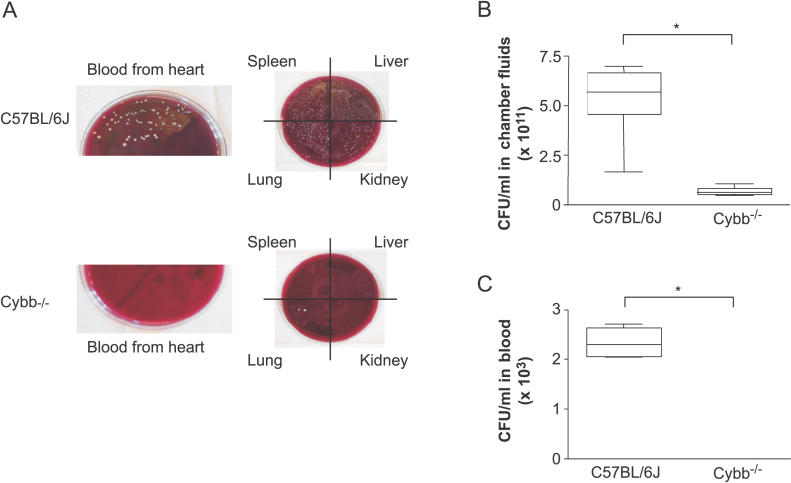
Bacteremia in Wild-Type and Cybb^−/−^ Mice 3 d after Challenge with the P. gingivalis Wild-Type Strain (A) Representative pictures of P. gingivalis colonies grown on anaerobic blood agar plates were taken using samples from the hearts, spleens, livers, lungs, and kidneys of C57BL/6J and Cybb^−/−^ mice, as indicated, 3 d after challenge with 100-μl volume, 1 × 10^9^ CFU/ml of P. gingivalis strain W83. (B) Increased numbers of P. gingivalis are present in the chamber fluid samples obtained from the C57BL/6J mice compared to the knockout Cybb^−/−^ mice. (C) In contrast to the C57BL/6J mice, no bacteria are detected in blood samples collected from the hearts of challenged Cybb^−/−^ mice. The values (CFU/ml) were determined by counting bacterial colonies on anaerobic agar plates (*n* = 3 mice). The data shown are the median (thick horizontal bars) and interquartile range for each group of mice. *p < 0.05; Mann-Whitney *U* test.

We have previously determined that the formation of skin lesions in C57BL/6J mice challenged with P. gingivalis indicates systemic spread of the bacterium [28]. This observation was confirmed in a separate experiment in which C57BL/6J and Cybb^−/−^ mice were killed on day 3 after inoculation with the wild-type P. gingivalis strain, and the numbers of bacteria present in the chamber fluid samples and blood drawn from the heart and extracts of the spleen, liver, lung, and kidney were determined (Figure 9). Every tissue examined from the C57/BL6J mice infected with the wild-type P. gingivalis strain was colonized with *P. gingivalis,* indicating that bacteremia led to the colonization of several organs (Figure 9A). The robust proliferation of P. gingivalis at these sites may have contributed to the observed mouse mortality following challenge with the wild-type P. gingivalis strain. In contrast, in the case of the Cybb^−/−^ mice, culturing of the blood, spleen, and liver extracts gave negative results, while only a few sporadic colonies were detected in the lung tissue extract (Figure 9A).

### The Exuberant Proliferation of P. gingivalis in Wild-Type Mice Is Unrelated to the Generation of Growth-Promoting Nutrients by the Oxidative Burst

**Figure 10:**
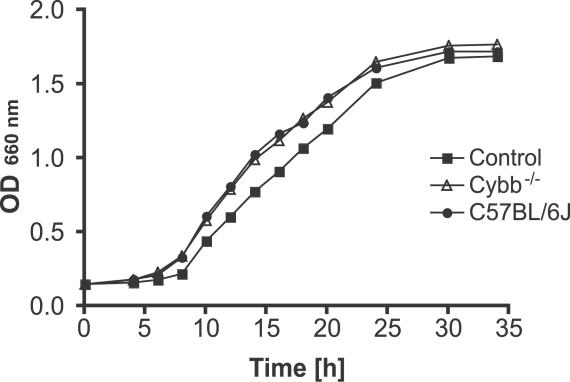
Kinetics of Wild-Type P. gingivalis Growth in Media Supplemented with Inflammatory Exudates Elicited in Wild-Type and Cybb^−/−^ Mice with Thioglycollate

To investigate the possibility that the presence of the NADPH oxidase in host cells allows more robust growth of P. gingivalis by providing essential growth factors derived from the breakdown products of oxidized proteins and fatty acids (e.g., C5a and arachidonate, respectively), we compared the levels of bacterial growth in media supplemented with the inflammatory exudates elicited from wild-type and Cybb^−/−^ mice by thioglycollate treatment. The growth of the wild-type P. gingivalis strain was similar in both media (Figure 10), which suggests that the systemic dissemination of P. gingivalis in wild-type mice is not due to NADPH oxidase–dependent generation of growth factors.

### Rbr Expression by P. gingivalis Leads to Increased Oral Bone Loss Following Infection

We also examined the pathogenic potential of the wild-type and Rbr^−^ P. gingivalis strains in a model of oral infection [50]. As expected, mice that were orally challenged with wild-type P. gingivalis exhibited significantly greater alveolar oral bone loss compared to unchallenged mice (Figure 10). In contrast, we did not observe significant changes in oral bone loss in wild-type mice infected with the P. gingivalis Rbr^−^ strain relative to the unchallenged wild-type mice. Thus, the presence of Rbr promotes increased P. gingivalis–induced inflammation and subsequent oral bone loss in this mouse model. However, in this case, and in resemblance to human periodontal disease, the infection was localized to the periodontal tissue. In contrast to the situation in subcutaneous chambers, the local host defense system and/or bacterial growth conditions in the oral cavity prevent systemic dissemination of *P. gingivalis.*

## Discussion

In this study, we demonstrate that bacterial Rbr protects in vivo against the host-mediated killing of the oral anaerobe, P. gingivalis*,* but that Rbr does not protect against the neutrophil oxidative burst. These conclusions are based on two observations. First, the wild-type and Rbr^−^ P. gingivalis strains did not differ in their susceptibilities to killing by normal neutrophils. Second, although the *rbr* mutation hindered the growth and virulence of P. gingivalis in mice, no difference was observed in vitro in terms of the killing of P. gingivalis between the normal and NADPH oxidase–deficient neutrophils.

Interestingly, the virulence of Rbr-deficient P. gingivalis was not restored in phagocytic Cybb^−/−^ mice. Exactly to the contrary, the genetic abrogation of the oxidative burst in Cybb^−/−^ mice was found to protect animals from mortality related to inoculation with wild-type *P. gingivalis.* Significantly, this protection was even more pronounced than in wild-type mice infected with the P. gingivalis Rbr^−^ mutant strain (Figure 1). As compared to the wild-type P. gingivalis strain, the isogenic Rbr^−^ mutant strain grew slower, reaching lower numbers in wild-type mice. Furthermore, the Rbr^−^ mutant strain was found in higher numbers in wild-type mice than in Cybb^−/−^ mice. In Cybb^−/−^ mice, the initial growth rate of the Rbr^−^ mutant was significantly reduced in comparison to that observed for the wild-type strain (Figure 2).

These interesting observations raise several important questions with regard to how Rbr protects *P. gingivalis.* From the data presented, and in agreement with previous reports showing no clear difference in P. gingivalis killing by neutrophils under aerobic and anaerobic conditions [31,51], it is obvious that both in vivo and in vitro Rbr is dispensable for protection against ROS generated by the phagocyte-derived oxidative burst. Physiologically, this is understandable since the ROS-quenching function of Rbr would be redundant within the anaerobic habitat of the subgingival bacteria plaque occupied by *P. gingivalis,* in which the oxygen-dependent bactericidal activities of neutrophils are nonfunctional [29]. In anoxic environments, such us deep wounds, periodontal pockets, and subcutaneous chambers, the major weapons used by phagocytes against invading bacteria are RNS generated by iNOS (NOS2) and/or bactericidal peptides and proteins, which expression is strongly enhanced in hypoxia [52]. The effectiveness of neutrophil-derived bactericidal peptides in the killing of P. gingivalis [30] and other bacteria [53] is well documented. Since RNS have been shown to possess strong antimicrobial activities against a broad array of bacteria [54], it is not surprising that RNS participate in P. gingivalis killing by murine macrophages (Figure 4). This ex vivo finding corroborates very well the observation that the presence of functional iNOS is essential to attenuate P. gingivalis growth in vivo in mice [33]. Significantly, this antibacterial effect was exerted in the anoxic environment of the subcutaneous chambers.

In as much as RNS participation in P. gingivalis killing was anticipated, the ability of Rbr to protect against the antibacterial activity of RNS was somewhat unexpected, making this a novel and interesting discovery. Significantly, the dual function of Rbr elicited protection of P. gingivalis in vivo not only against oxygen stress but also against RNS correlates well with the pathological events associated with the infection. Taking into account that mice with the genetically inactivated oxidative burst produce greater amounts of RNS in vivo than normal animals [55–57], our findings explain very well the observed differences in pathology and microbial growth of the P. gingivalis strains in normal and Cybb^−/−^ mice. In wild-type mice, the initial production of ROS in a chamber, before the environment becomes anoxic, may suppress RNS generation and allow robust growth of the Rbr-protected *P. gingivalis* strain, but not the Rbr^−^ mutant (Figure 2). In Cybb^−/−^ animals, the growth of the wild-type P. gingivalis strain is controlled by the elevated level of RNS, which overwhelms the protection afforded by Rbr. Finally, in concordance with our findings, the Rbr^−^ P. gingivalis strain in the absence of the phagocyte oxidative burst showed the highest level of growth attenuation. In vivo, the predominant sources of antibacterial RNS are stimulated monocytes, macrophages, and epithelial cells, but not neutrophils [58,59]. In this context, the finding that the role of Rbr in P. gingivalis infection is related to protection against RNS, but not ROS, explains why the presence or absence of this protein has no effect on bacterium killing by neutrophils in vitro, although Rbr shows protective functions in vivo.

In addition to facilitating the growth of *P. gingivalis,* the combination of the host phagocyte oxidative burst and the expression of bacterial Rbr appears to be instrumental in P. gingivalis systemic dissemination from the subcutaneous chambers, which is manifested as bacteremia and heavy colonization of the spleen, liver, lungs, and kidneys (Figure 9). We observed very limited dissemination of the P. gingivalis wild-type strain in the Cybb^−/−^ mice, apparently due to enhanced production of RNS in the oxidative burst–deficient animals [55–57]. Interestingly, significantly higher numbers of dead cells were found in the chamber fluids recovered from Cybb^−/−^ mice than from wild-type animals (Figure 7B). A possible reason for this finding is the very complex intimate relationship between nitric oxide and superoxide in leukocyte migration, cell survival, cell apoptosis, and clearance of apoptotic and necrotic cell bodies [60]. In this context, elimination of the phagocyte NADPH oxidase–dependent regulatory functions may have profound effects on cell fate at the site of a confined local infection [61–66], resulting in disproportionately high numbers of dead cells in the chamber fluids from Cybb^−/−^ mice.

The other striking difference in the contents of the chamber fluid samples was the level of MPO, an indicator of neutrophil turnover, which was highest in wild-type mice infected with the P. gingivalis wild-type strain (Figure 7C). Furthermore, this particular combination of host and pathogen was accompanied by a significant increase in the numbers of circulating neutrophils and considerable systemic activation of these neutrophils, as illustrated by the two-fold decrease in L-selectin expression on peripheral neutrophils [67] (Figure 8) and the high level of MPO in the serum. Finally, MPO was detected in the serum samples, albeit at very low levels, of Cybb^−/−^ mice inoculated with the wild-type strain (Figure 7C). The presence of circulating MPO can be directly linked to the pathology of P. gingivalis infection in wild-type mice, since this protein by binding to CD11b/CD18 integrins serves as a powerful autocrine and paracrine stimulator of neutrophil activation, provoking degranulation, expression of integrins, and oxidative burst discharge [48]. Collectively, these data indicate that the functional oxidative burst response and neutrophil activation are prerequisites for P. gingivalis to escape from the chambers and disseminate to the organs. In this context, it is important to reiterate that the differences noted in the growth rate and dissemination of P. gingivalis in wild-type and Cybb^−/−^ mice were not related to differences in the availability of nutrients or growth factors generated through the oxidation of some host components (Figure 10). These results contrast with those reported previously for aerobic pathogens, such as *Burkholderia cepacia, Staphylococcus aureus, Klebsiella pneumoniae, Escherichia coli,* and *Salmonella typhimurium,* all of which have been shown to exhibit enhanced virulence in genetically abrogated phagocyte oxidative burst animal models [68–72].

The mortality rates of mice infected with P. gingivalis correlated with increases in the concentration of LPS (in the serum and chamber fluid samples) and levels of neutrophil activation in the blood samples (Figure 5). The IL-6 and TNF-α concentrations in the sera were unrelated to the LPS levels, either in the chamber fluid samples or in blood (Figure 6), which is not surprising given that P. gingivalis LPS is known be nontoxic and a weak stimulator of cytokine release [73]. Thus, animal mortality following P. gingivalis infection was not due to a “cytokine storm,” as in the case of sepsis induced by enterobacterial LPS [74]. These results re-emphasize the self-destructive function of the oxidative burst aimed at *P. gingivalis.* In addition to killing microbes, the oxidative burst may have important regulatory functions, including effects on neutrophil gene transcription [75] and macrophage and lymphocyte activation [76]. A bacterium-induced malfunction of these regulatory pathways and excessive oxidative burst and neutrophil degranulation in aerated tissues, such as the lungs and tissues supplied with oxygen by the blood, which include the spleen, liver, and kidneys, all of which were heavily colonized by P. gingivalis in the wild-type mice (Figure 9A), may cause serious loss of function of these organs and animal death. It is tempting to speculate that P. gingivalis subverts these signaling pathways to its advantage, such that in Cybb^−/−^ mice the virulence of the organism is attenuated.

Impaired neutrophil chemotaxis [77,78] and deficiency of serine proteases [79] or the bactericidal peptide LL-37 [80] in neutrophil granules are associated with aggressive forms of periodontal disease despite the presence of a normal oxidative burst. Conversely, patients with CGD are devoid of the NADPH oxidase activity and seldom suffer from periodontal disease, although they are prone to severe recurrent bacterial infections [10]. In addition, recent evidence indicates that a molecular defect in intracellular lipid signaling contributes to excessive superoxide generation by neutrophils leading to tissue destruction in patients with periodontal disease [81]. These clinical associations, which are in concordance with our data, negate the importance of the oxidative burst in combating periodontal pathogens. Collectively, these results suggest that the host respiratory burst response to acute anaerobic infection may contribute significantly to the morbidity associated with periodontal disease.

**Figure 11:**
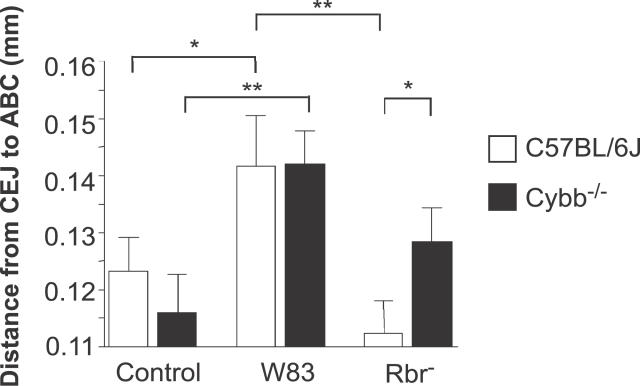
The Presence of the Rbr Gene Increases P. gingivalis–Induced Oral Bone Loss Following a 10-d regimen of oral trimethoprim-sulfamethoxazole and a 3-d rest period, mice were administered 1.0 × 10^9^ CFU of the P. gingivalis strains by oral gavage three times over a 1-wk period. One group of animals was not treated, and served as the age-matched controls. Six wk after oral challenge, all of the animals were killed. Linear bone loss measurements (*n* = 14) were obtained from the maxillary molars of each mouse, and the data for linear bone loss (in mm) from the cemento-enamel junction to the alveolar bone crest are presented as the means ± SEM of the pooled linear measurements obtained for each group. *p < 0.05; **p < 0.01; as determined by the Student's *t* test.

The insignificance of the oxygen-dependent bactericidal activity in maintaining periodontal tissue homeostasis following infection with anaerobic pathogens is also clear from our experimental data obtained using the oral model for P. gingivalis infection (Figure 11). The levels of oral bone loss induced by the wild-type P. gingivalis strain were similar in the C57BL/6J and Cybb^−/−^ mice. Unlike subcutaneous chamber inoculation, the presence of the intact oxidative burst system in mice orally infected with the wild-type P. gingivalis strain did not result in this model in excessive inflammation and subsequent bone loss or systemic dissemination of infection and high mortality. This is apparently due to a localized type of periodontal infection, in which P. gingivalis colonizes only subgingival tooth surfaces, growing in the form of a biofilm separated from the circulation by a thick “wall” of leukocytes [82]. This protection, together with defenses in the blood, is evidently sufficient since human clinical cases of bacteremia caused by P. gingivalis are unknown. For *P. gingivalis,* Rbr obviously plays a role in shielding the bacterium against the host immune system at periodontal sites, since the presence of Rbr contributed to the morbidity induced by P. gingivalis infection. This is apparent from the fact that in normal mice, oral bone loss was significantly greater upon infection with the wild-type P. gingivalis strain as compared to that observed following infection with the Rbr^−^ mutant strain (Figure 11).

The consequences of the harmful interaction between the host oxidative burst response and anaerobic bacteria possessing Rbr are likely to extend beyond infection by the periodontal disease pathogen *P. gingivalis.* A recent BLAST search of the 878 genomic sequences in the National Center for Biotechnology Information Microbial Genomes Database with the *P. gingivalis rbr* gene found 114 homologs in 69 organisms, the vast majority being obligate anaerobes or microaerophiles. Although the majority of these air-sensitive bacteria are not known to be harmful to humans, 16 are human commensals or pathogens. These include B. pseudomallei, a causative agent of melloidosis and a potential bioterrorist weapon [83], *B. cepacia,* an opportunistic colonizer of the lungs of patients with cystic fibrosis [84], *Campylobacter jejuni,* the most common cause of bacterial enterocolitis in humans [85], and Clostridium tetani and *C. perfringens,* which are the causative organisms of tetanus and gas gangrene, respectively. Furthermore, human commensals that carry the *rbr* gene homolog, such as *Clostridium, Bacteroides, Prevotella, Treponema,* and *Fusobacterium* species, are the most frequently isolated organisms in anaerobic bacteremia, endocarditis, and bone and joint infections, as well as skin and oral abscesses [86]. Thus, our results on P. gingivalis may have relevance to oxygen-dependent killing mechanisms operating during anaerobic infections and the contribution of Rbr to protection against molecules of host defense by other pathogenic anaerobic organisms. In the clash between these two systems, an oxidative burst elicited by neutrophils, which are unable to eliminate the bacteria, may be responsible for host tissue damage, either directly through oxidative destruction of macromolecules or indirectly, by activation of other cells. This leads to systemic bacterial dissemination, organ colonization and injury, and eventually death of the host.

## Materials and Methods

### Mouse strains.

All of the animal experiments were performed in accordance with the guidelines of the Institutional Animal Care and Use Committee of Boston University and in conformance to the Standards of Public Health Service Policy on Human Care and Use of Laboratory Animals. Six-week-old female C57BL/6J and phagocytic Cybb^−/−^ mice were purchased from the Jackson Laboratory (Bar Harbor, Maine, United States). All of the animals received the standard laboratory chow diet and were examined daily for any adverse health conditions.

### P. gingivalis cultures.

P. gingivalis strain W83 was grown in anaerobe broth (Difco Laboratories, Sparks, Maryland, United States) in an anaerobic chamber in an atmosphere of 80% N_2_, 10% H_2_, and 10% CO_2_. The isogenic *P. gingivalis rbr*-knockout strain (Rbr^−^) [22] was grown under the same conditions with the addition of erythromycin at a concentration of 5 μg/ml. Bacterial cell counts were determined on all the P. gingivalis cultures prior to the animal experiments, to confirm viability and to determine the number of viable organisms present at the time of animal challenge.

### Chamber implantation.

Following anesthesia with isoflurane, sterile wire, coil-shaped stainless steel chambers were implanted subcutaneously in the dorso-lumbar region of each mouse [27]. Incisions were closed using 4.0-G silk sutures and the animals were allowed to rest for 14 d, at which timepoint all the incisions had healed completely.

### Chamber inoculation with P. gingivalis.

C57BL/6J and Cybb^−/−^ animals were each divided into three groups, with *n* = 7 for each group. The first group was inoculated with 0.1 ml of P. gingivalis W83, the second group received 0.1 ml of the Rbr^−^ strain (10^9^ CFU/ml), and the third group was inoculated with vehicle only. A minimum of 70 μl of chamber fluid was collected from each mouse using a syringe with a 25-G sterile hypodermic needle at 1, 3, 6, and 10 d after challenge.

### Chamber fluid analysis.

Each chamber fluid sample was divided into several samples to be used for the determination of CFU/ml (10 μl), total inflammatory cell counts (10 μl), and differential inflammatory cell counts (5 μl); the remainder (~40 μl) was stored at −80 °C for subsequent enzyme-linked immunosorbent assay (ELISA) determinations of the IL-6, TNF-α, IL-10, LPS, and MPO levels.

### Microbial analysis**.**

Aliquots of chamber fluid from each mouse (10 μl) were serially diluted ten-fold with 1% peptone. Dilutions were plated onto anaerobic blood agar plates (Remel, Lenexa, Kansas, United States) in duplicate and incubated in an anaerobic chamber for 6–7 d. The CFU/ml values were determined by direct colony counting. The presence of P. gingivalis was confirmed by Gram staining and colony PCR. The forward (sense) primer 5′-CGTGCCAGCAGCCGCGGTAATACG-3′ and the reverse (antisense) primer 5′-TACATAGAAGCCCCGAAGGAAGAC-3′) were used to amplify the P. gingivalis 16S gene. PCR amplification was carried out using the following conditions: 40 cycles of denaturation at 95 °C for 30 sec, annealing at 60 °C for 30 sec, and elongation at 72 °C for 1 min. The PCR products were separated by agarose gel electrophoresis and visualized using a UV transilluminator after staining with ethidium bromide.

### Killing of bacteria by murine bone marrow and human peripheral blood neutrophils.

Human neutrophils were purified from fresh human blood by dextran sedimentation and centrifugation through Ficoll/Hypaque. The mouse neutrophils were isolated from the bone marrow. Briefly, the bone marrow was collected from the femurs and tibias of normal and Cybb^−/−^ mice. The distal and proximal tips were removed and washed with cold KRG buffer (120 mM NaCl, 5 mM KCl, 8.3 mM Na_2_HPO_4_ [pH 7.3], 10 mM glucose, 1 mM CaCl_2_) using a 1-ml syringe and 27-G needle. After dispersing cell clumps using a 1-ml pipette on ice, the suspension was centrifuged (200*g,* 10 min, 4 °C) and the cell pellet was resuspended in 2 ml of sterile PBS. The cells were carefully layered on the top of a discontinuous Percoll gradient (72%, 63%, 50%; 2 ml each). After centrifugation (500*g,* 30 min), the lowest band containing neutrophils was collected, residual erythrocytes were removed by hypotonic shock, and the neutrophils were suspended in PBS at a concentration of 2 × 10^7^ cell/ml.

Mouse (three to five animals) and human (one donor) neutrophils (2.5 × 10^7^) in 0.5 ml were mixed with IgG-opsonized P. gingivalis (5 × 10^6^ CFU/ml) at a ratio of one target organism to five neutrophils in 0.5 ml PBS and incubated either in an aerobic or anaerobic atmosphere. The rate of bacterial killing was measured as described previously [87], omitting lysostaphin. Cell lysates that contained viable P. gingivalis were plated on 10% horse blood agar plates and incubated anaerobically for 5 d. The results were calculated at the mean (±SE) from at least two experiments with colony counts performed in triplicate for each sample and expressed as a percentage of the original numbers at time zero.

### Killing of *P.gingivalis* W83 and Rbr^−^ strain by murine macrophages in the presence of L-NAME.

Mice were injected intraperitoneally with sterile thioglycollate broth (3%; Difco Laboratories) at 3 ml/mice, and inflammatory exudates were recovered after 72 h by peritoneal lavage with sterile RPMI 1640 medium supplemented with 10% FCS. Cells harvested from six animals were pooled, washed in RPMI 1640 with 10% FCS and seeded on 24-well plates (1 × 10^6^/well; TPP Switzerland, Trasadingen, Switzerland) in 1 ml of RPMI 1640 with 10% of FCS, and divided into two groups. After 6 h, both groups were stimulated to express iNOS by the addition of E. coli serotype O26:B6 LPS (400 ng/ml; Sigma, Saint Louis, Missouri, United States). One group was exposed to LPS in the presence of L-NAME (Calbiochem, San Diego, California, United States) at a final concentration of 5 mM. After overnight culture, the nitrite and nitrate levels were determined by the Griess reaction. To synchronise phagocytosis, plates that contained macrophages were inoculated with 5 × 10^6^ fresh, opsonized bacteria per well and centrifuged at 380*g* for 8 min at 4 °C. At the indicated timepoints, the supernatant and cells were harvested, lysed, and plated onto blood agar plates. Colonies were counted after 5 d of culture, and the percentage of P. gingivalis survival was calculated according to the equation (CFU at *t* / CFU at *t_0_*) × 100. This assay measures the total numbers of uningested (in the supernatant) and ingested (in the cell lysate) viable bacteria.

### Assessment of ROS production by bone marrow neutrophils exposed to *P. gingivalis.*

Bone marrow neutrophils from four mice (1.0 × 10^7^/ml) suspended in sterile PBS were seeded in 100 μl into a black 96-well plate (Nunc, Rochester, New York, United States). Then, 100 μl of serum-opsonized P. gingivalis W83 or Rbr^−^ (5.0 × 10^8^/ml) was added together with 50 μl of luminol solution (0.53 mg/ml; Sigma) and chemiluminescence was recorded for 120 min using the EG&G Berthold MicroLumet LB 96P luminometer (Berthold Technologies, Bad Wildbad, Germany).

### Analysis of P. gingivalis growth in media supplemented with an inflammatory exudate from wild-type and Cybb^−/−^ mice.

Mice were injected intraperitoneally with sterile thioglycollate broth (3%; Difco Laboratories) at 3 ml/mice and inflammatory exudates were recovered by peritoneal lavage with sterile RPMI 1640 medium supplemented with 10% FCS. The lavage was sonicated to disintegrate the cells, mixed 1:1 with a two-fold dilution of Scheadler broth, filter-sterilized (0.4-μm filter) and inoculated with *P. gingivalis.* Bacterial growth under anaerobic conditions was monitored by measurement of the culture turbidity at 660 nm in a spectrophotometer.

### Analysis of blood neutrophil activation.

Subcutaneous chambers were implanted in the C57BL/6J mice. Ten d later, the chambers were inoculated with 100 μl of P. gingivalis W83 or Rbr^−^ strain (1 × 10^9^/ml). As a control, 100 μl of sterile PBS was inoculated into the chamber. Three d after inoculation, blood samples were collected from the heart into heparinized tubes, and the blood cells were stained with FITC-conjugated Ly-6G monoclonal antibodies and R-phycoerythrin–conjugated CD62L monoclonal antibodies. Rat IgG_2A_ κ monoclonal antibodies were used as the isotype control. The samples were analyzed by flow cytometry in the FACSCalibur (BD Biosciences, Franklin Lakes, New Jersey, United States) using the CellQuest Software (BD Biosciences).

### Inflammatory cell analysis.

A 10-μl chamber fluid sample from each mouse was stained with trypan blue, and total and viable inflammatory cell counts were obtained using light microscopy at 40× magnification in a hemocytometer chamber. In addition, 5-μl samples were placed on slides and stained with Giemsa-Wright stain for differential cell counts. The neutrophils were differentiated based on their lobed nuclei.

### Quantification of cytokine levels.

The serum and chamber fluid levels of TNF-α, IL-6, and IL-10 were determined by ELISA (Pierce Endogen, Rockford, Illinois, United States). For the determination of LPS levels, the QCL-1000 Chromogenic LAL Test Kit (Cambrex, Santa Rosa, California, United States) was used. The levels of MPO in the sera and chamber fluids were determined by ELISA (Calbiochem, La Jolla, California, United States).

### Assessment of oral bone loss.

Oral bone loss was assessed at defined landmark sites on the maxillary molars of each mouse, as previously described [50]. Using a stereomicroscope, an observer who was blinded to the identity of the groups obtained linear measurements (14 sites) for each animal from the cemento-enamel junction to the alveolar bone crest [88]. Measurements were obtained with a digital camera linked to a computer, and onscreen measurements were converted to micrometers using IPLabs (Scanalytics Inc., Fairfax, Virginia, United States).

### Statistical analysis.

Statistical comparison analyses were performed using the Mann-Whitney *U* test or Student's *t* test as indicated in the figure legends. Differences in the data were considered significant when the probability value was less than 5.0% (*p* < 0.05).

## Supporting Information

### 

#### Accession Numbers.

The National Center for Biotechnology Information (NCBI) (http://www.ncbi.nlm.nih.gov) accession number for Rbr is AAK19552.

The Institute for Genomic Research–Comprehensive Microbial Resource database (TIGR-CMR) (http://cmr.tigr.org/tigr-scripts/CMR/CmrHomePage.cgi) accession number for the *P. gingivalis rbr* gene is PG 0195.

## Abbreviations

CGD: chronic granulomatous disease
Cybb^−/−^: NADPH oxidase–null
ELISA: enzyme-linked immunosorbent assay
iNOS: inducible nitric oxide synthase
LPS: lipopolysaccharide
MPO: myeloperoxidase
Rbr: rubrerythrin
RNS: reactive nitrogen species
ROS: reactive oxygen species
